# Key pathways and genes in hepatitis B virus-related liver inflammation: Expression profiling and bioinformatics analysis

**DOI:** 10.1097/MD.0000000000030229

**Published:** 2022-08-26

**Authors:** Jing-Yuan Zhao, Zhao-Zhong Zhong, Li-Yun Zhao, Wen Li

**Affiliations:** a Department of Hepatobiliary Surgery, The Second Affiliated Hospital of Nanchang University, Nanchang, Jiangxi, China; b Department of Endocrinology, The First Hospital of Nanchang, Nanchang, China; c Department of Hepatobiliary Surgery and Guangdong Key Laboratory of Liver Disease, Third Affiliated Hospital of Sun Yat-sen University, Guangzhou, Guangdong, China.

**Keywords:** bioinformatics, differentially expressed genes, hepatitis B virus, hepatocellular carcinoma, hub genes, liver inflammation

## Abstract

Chronic hepatitis B virus infection has become a major public health issue worldwide, which can lead to liver inflammation, fibrosis, and hepatocellular carcinoma. According to the inflammation activity, liver tissues can be divided into 5 grades (G0–G4). However, the mechanism of the development of liver inflammation remains unclear. In our study, expression profiling by microarray and bioinformatics technology was used to systemically identify differentially expressed genes (DEGs) between low grades (G0–G1) and high (G2–G4) grades of liver inflammation. Gene Ontology (GO) enrichment analysis, Kyoto Encyclopedia of Genes and Genomes pathway enrichment analysis, and protein–protein interaction network construction were performed for further identification of the key functions, pathways, and hub genes that might play important roles in the inflammation development. A total of 1982 DEGs were identified, consisting of 1220 downregulated genes and 762 upregulated genes. GO analysis revealed the DEGs were mainly enriched in GO terms that related to neutrophil activation and degranulation. MAPK1, ITGA2, CDK2, TGFB1, CDKN2A, MTOR, IL6, PCNA, OAS2, and EP300 were hub genes that had the highest centricity and might be potential markers for inflammation development. This study identified the differentially expressed genes between different grades of inflammation, which would enlighten the study that focuses on the mechanism of liver inflammation development.

## 1. Introduction

Chronic hepatitis B virus (HBV) infection has become a major public health issue worldwide.^[1–3]^ The rate of HBV infection has remained stubbornly high, especially in China. It is reported that hepatitis B carriers accounted for about 10% of the total population in China.^[4]^ Persistent HBV infection can lead to hepatic inflammation, fibrosis, hepatocellular carcinoma (HCC), and increased mortality.^[5,6]^ According to the inflammation activity, liver tissues can be divided into 5 grades (G0–G4). For patients with grade ≥2, antiviral treatment is recommended to reduce the progression of the disease.^[7]^ Our previous study also showed that the development of inflammation would bring up the risk of HCC.^[8,9]^ However, the mechanism of the development of liver inflammation activity remains unclear.

Recently, a number of studies have investigated the pathological mechanisms of hepatic inflammation progression. Firstly, the recruitment of immune cells to the liver was considered essential for the initiation of HBV-related hepatic inflammation and subsequent chronic persistent infection.^[10,11]^ The different expression levels of cytokines were also discussed in different phases of HBV infection.^[12,13]^ For example, Interleukin-22 is a cytokine that is involved in the pathogenesis of the liver disease but had a controversial role in liver inflammation in patients with HBV infection.^[14,15]^ Secondly, endoplasmic reticulum and mitochondrial dysfunctions were associated with the pathogenesis of hepatic inflammation, leading to liver injury.^[16,17]^ Thirdly, structural changes in the gut microbiota, bacterial translocation, and the resulting immune injury might affect the occurrence and development of liver inflammation caused by chronic HBV infection.^[18]^ However, other mechanisms associated with hepatic inflammation have not been identified. Therefore, further research is needed to reveal other potential mechanisms and identify target genes for the treatment of HBV-related hepatic inflammation.

In our study, expression profiling by microarray and bioinformatics technology was used to systemically identify differentially expressed genes (DEGs) between low grades (G0–G1) and high (G2–G4) grades of liver inflammation. Gene Ontology (GO) enrichment analysis, Kyoto Encyclopedia of Genes and Genomes(KEGG) pathway enrichment analysis, and protein–protein interaction (PPI) network construction were performed for further identification of the key functions, pathways, and hub genes that might play important roles in the inflammation development. The results will bring insight into the mechanism of liver inflammation development and may provide a theoretical basis and diagnostic markers for HBV-related hepatic inflammation.

## 2. Results

### 2.1. Identification of DEGs

**Figure 1.**
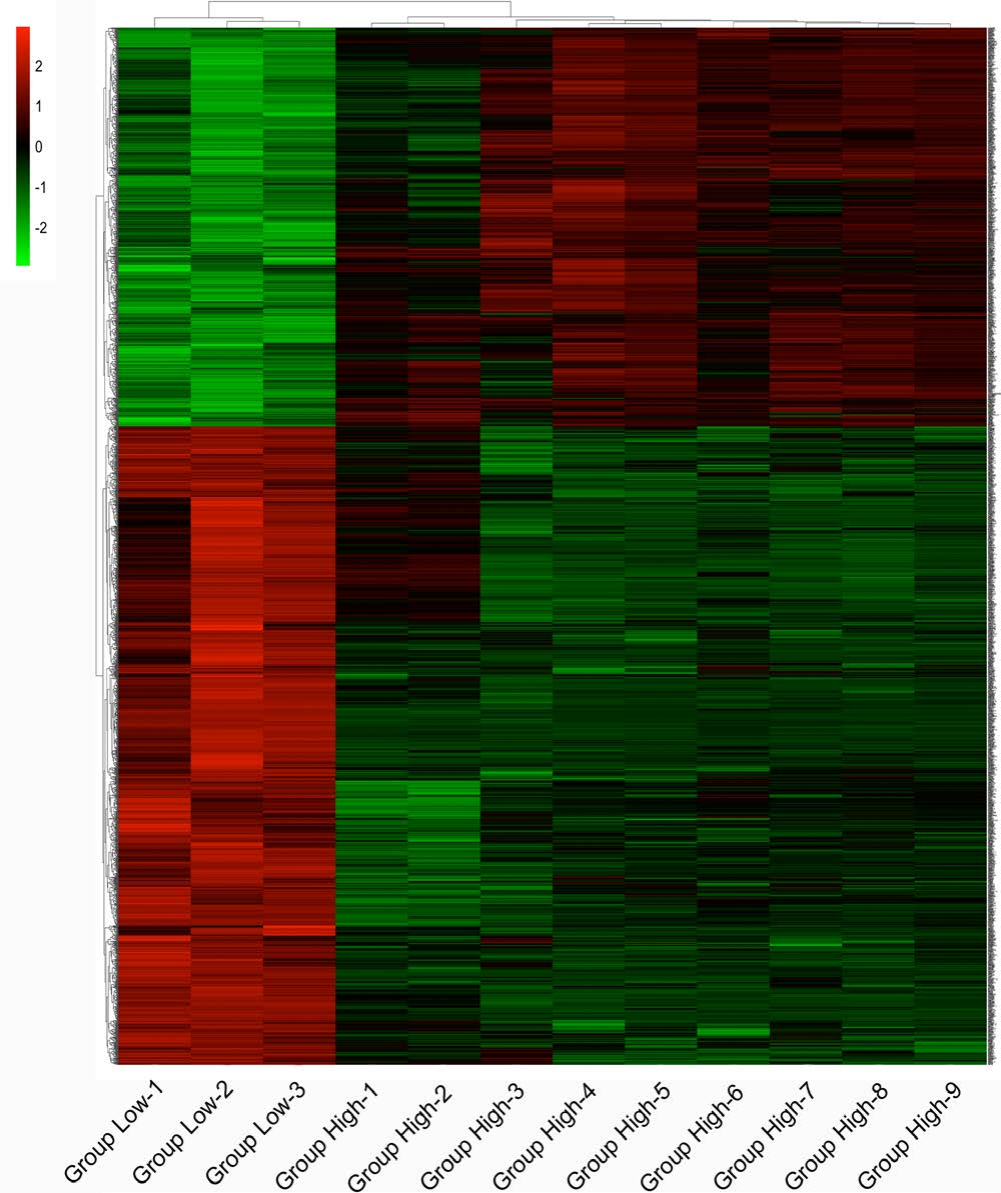
Heat map of differentially expressed genes between Group Low and Group High. Green represents lower expression levels, red represents higher expression levels, and black represents that there are no differences in expression among the genes.

After standardization of the microarray results, 1982 DEGs were identified, consisting of 1220 downregulated genes and 762 upregulated genes between Group Low and Group High. The heat map showed clustering of DEGs between the 2 groups. In clustering analysis, upregulated and downregulated genes are colored in red and green, respectively (Fig. 1).

### 2.2. KEGG and GO enrichment analyses of DEGs

To analyze the biological classification of DEGs, functional and pathway enrichment analyses were performed by the Clusterprofiler package for upregulated and downregulated DEGs, respectively.

Referring to the GO terms of biological processes (BP) category, upregulated DEGs were significantly enriched in neutrophil activation, granulocyte activation, leukocyte degranulation, neutrophil activation involved in immune response, myeloid cell activation involved in immune response, myeloid leukocyte, mediated immunity, neutrophil degranulation, and neutrophil mediated immunity (Table 1). This result might indicate that neutrophil activation and degranulation play an important role in liver inflammation development. Referring to the cellular component (CC) category, upregulated DEGs were mainly enriched in azurophil granule lumen, secretory granule lumen, cytoplasmic vesicle lumen, and vesicle lumen in the CC component category (Table 1A). Azurophil granules are specialized lysosomes of the neutrophil and contain at least 10 proteins implicated in the killing of microorganisms. It might indicate that neutrophils may have higher antimicrobial activity in liver tissues with higher inflammation grades. Despite the significant enrichment in CC and BP categories, upregulated DEGs did not significantly enriched in any molecular function (MF) category. Downregulated DEGs were enriched in GO terms of BP category, including negative regulation of cell adhesion and muscle cell proliferation, but were not significantly enriched in any GO terms of the CC or MF categories (Table 1B).

**Table 1:** GO enrichment analysis of DEGs.

Term	Description	Count in gene set	*P* value
**A. Upregulat0ed**			
GO-BP			
GO:0042119	Neutrophil activation	54	9.41E − 10
GO:0036230	Granulocyte activation	54	1.44E − 09
GO:0043299	Leukocyte degranulation	55	3.49E − 09
GO:0002283	Neutrophil activation involved in immune response	52	3.76E − 09
GO:0002275	Myeloid cell activation involved in immune response	55	6.07E − 09
GO:0002444	Myeloid leukocyte-mediated immunity	55	8.27E − 09
GO:0043312	Neutrophil degranulation	51	8.79E − 09
GO:0002446	Neutrophil-mediated immunity	52	9.27E − 09
GO-CC			
GO:0035578	Azurophil granule lumen	15	2.06E − 05
GO:0034774	Secretory granule lumen	33	2.46E − 05
GO:0060205	Cytoplasmic vesicle lumen	34	2.93E − 05
GO:0031983	Vesicle lumen	34	3.14E − 05
**B. Downregulated**			
GO-BP			
GO:0007162	Negative regulation of cell adhesion	40	1.12E − 06
GO:0033002	Muscle cell proliferation	30	1.41E − 06

Terms represent the identification number of GO terms; description represents the names of GO terms; counts represent the number of genes enriched in GO terms.

BP = biological process, CC = cellular component, DEGs = differentially expressed genes, GO = Gene Ontology, MF = molecular function.

KEGG enrichment analysis revealed that the upregulated DEGs were mainly enriched in Graft-versus-host disease, Rheumatoid arthritis, Leishmaniasis, Influenza A, Type I diabetes mellitus, Hematopoietic cell lineage, Allograft rejection, Natural killer cell-mediated cytotoxicity, and Cytosolic DNA-sensing pathway, while downregulated DEGs were not enriched in any pathway (Table 2).

**Table 2:** KEGG pathway enrichment analysis of DEGs.

Upregulated			
Term	Description	Count in gene set	*P* value
hsa05332	Graft-vs-host disease	11	1.15E − 06
hsa05323	Rheumatoid arthritis	16	1.87E − 06
hsa05140	Leishmaniasis	14	3.87E − 06
hsa05164	Influenza A	22	6.70E − 06
hsa04940	Type I diabetes mellitus	10	1.43E − 05
hsa04640	Hematopoietic cell lineage	15	2.29E − 05
hsa05330	Allograft rejection	9	3.31E − 05
hsa04650	Natural killer cell-mediated cytotoxicity	17	6.78E − 05
hsa04623	Cytosolic DNA-sensing pathway	11	9.24E − 05

The term represents the identification number of the KEGG pathway; description represents the name of the KEGG pathway; and count in the gene set represents the number of genes enriched in the KEGG pathway.

DEGs = differentially expressed genes, KEGG = Kyoto Encyclopedia of Genes and Genomes.

### 2.3. PPI network construction and module analysis

**Figure 2.**
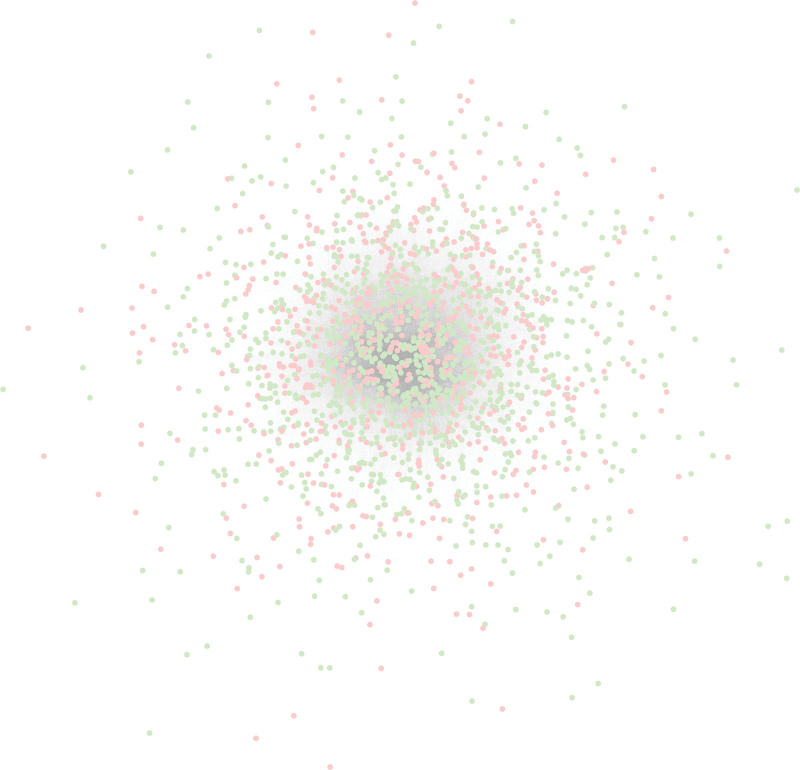
Protein–protein interaction network of differentially expressed genes between Group Low and Group High. Red nodes represent upregulated genes; green nodes represent downregulated genes.

**Figure 3.**
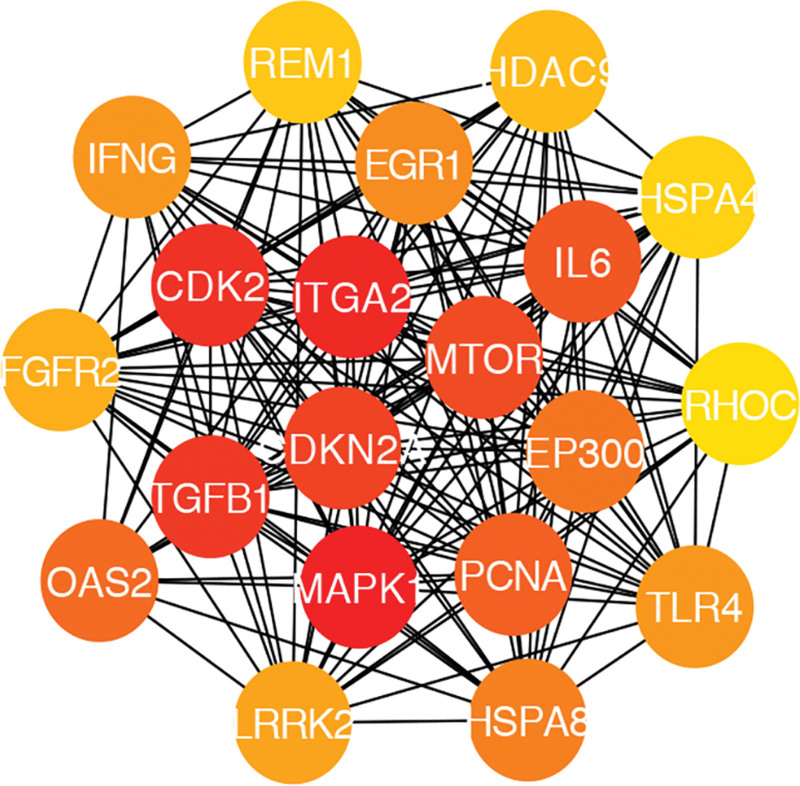
Protein–protein interaction network of top 20 genes in degree. Red represents higher degree and yellow represents lower degree.

Based on the STRING database, a total of 1920 nodes and 54,492 protein pairs were obtained. The PPI network of DEGs was constructed (Fig. 2). Genes that ranked top 20 in degree, closeness, and betweenness were displayed in Table 3. The network of these genes with top 20° was constructed (Fig. 3). A number of protein families, such as MAPK1, ITGA2, CDK2, TGFB1, CDKN2A, MTOR, IL6, PCNA, OAS2, and EP300 were selected as hub nodes based on degree ≥350. As can be seen in the table, these hub genes also ranked top 20 in closeness and betweenness.

**Table 3:** Nodes with top 20 values in closeness centrality, betweenness centrality, and degree centrality.

Nodes	Closeness	Nodes	Betweenness	Nodes	Degree
MAPK1	1.18E + 03	ITGA2	1.04E + 05	MAPK1	467
CDK2	1.17E + 03	CDK2	7.60E + 04	ITGA2	463
ITGA2	1.17E + 03	OAS2	6.35E + 04	CDK2	460
TGFB1	1.15E + 03	MAPK1	6.25E + 04	TGFB1	423
CDKN2A	1.15E + 03	TGFB1	6.08E + 04	CDKN2A	415
MTOR	1.14E + 03	IFNG	5.35E + 04	MTOR	391
PCNA	1.12E + 03	CDKN2A	5.14E + 04	IL6	369
IL6	1.12E + 03	SUMO2	4.73E + 04	PCNA	362
OAS2	1.11E + 03	MTOR	4.69E + 04	OAS2	355
EP300	1.11E + 03	SNCA	4.66E + 04	EP300	350
HSPA8	1.11E + 03	EP300	4.29E + 04	HSPA8	344
EGR1	1.11E + 03	HSPA8	3.88E + 04	EGR1	337
IFNG	1.10E + 03	PCNA	3.65E + 04	IFNG	336
TLR4	1.10E + 03	IL6	3.56E + 04	TLR4	336
LRRK2	1.10E + 03	GART	3.55E + 04	LRRK2	330
REM1	1.10E + 03	DNM2	3.46E + 04	FGFR2	321
HDAC9	1.09E + 03	HSPA4	3.43E + 04	HDAC9	320
HSPA4	1.09E + 03	CDH1	3.38E + 04	REM1	319
FGFR2	1.09E + 03	WDTC1	3.22E + 04	HSPA4	317
CDH1	1.09E + 03	TLR4	3.22E + 04	RHOC	313

**Figure 4.**
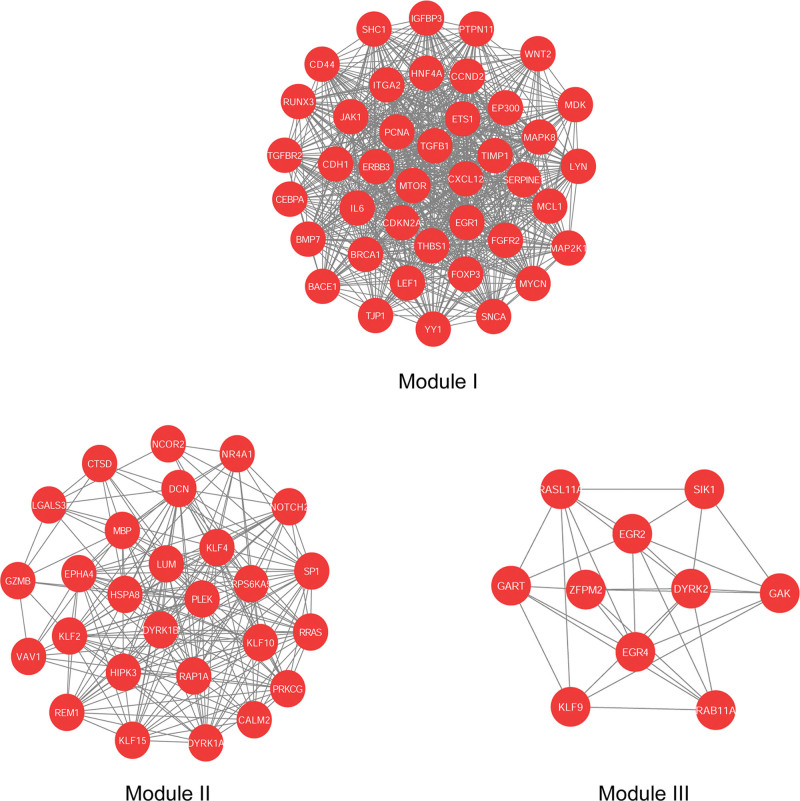
Three most significant modules. They are identified from the protein–protein interaction network using the molecular complex detection method with a score of > 6.0. Module 1: MCODE score = 35.85; Module 2: MCODE score = 15.692; Module 3: MCODE score = 6.889.

The most significant modules were obtained using MCODE. In total, 3 modules (Modules I, II, and III) were detected with a score >5, K-core= = 4 by MCODE (Fig. 4). As shown in Fig. 4, hub genes, including ITGA2, TGFB1, CDKN2A, MTOR, IL6, PCNA, and EP300 were also centered in module I, which had the highest score among these 3 modules. This might implicate these genes have had the highest centricity and may be the key genes and play important roles in liver inflammation development.

## 3. Discussion

In the last few years, many studies have focused on the mechanism of transformation from HBV-related hepatic inflammation to HCC.^[19–21]^ However, the mechanism of hepatic inflammation development had rarely been discussed. Present studies mainly focused on 1 or 2 individual genes and lacked a systematic identification of different expressions between different grades of inflammation.

In our study, expression profiling by microarray and bioinformatics technology was used to systemically identify DEGs between low grades and high grades of liver inflammation. GO and KEGG enrichment analyses were performed for further identification of the key functions,^[22]^ and pathways that might play important roles in inflammation development. PPI network construction and module analysis were performed to identify the hub genes.^[23]^

GO analysis results showed that upregulated DEGs were significantly enriched in neutrophil activation, granulocyte activation, leukocyte degranulation, neutrophil activation involved in immune response, myeloid cell activation involved in immune response, myeloid leukocyte, mediated immunity, neutrophil degranulation, and neutrophil-mediated immunity referring to BP category, which indicated that neutrophils activation and degranulation might play an important role in liver inflammation development. This is consistent with the previous study that the recruitment of immune cells is important in hepatic inflammation.^[24]^ Upregulated DEGs were mainly enriched in azurophil granule lumen, secretory granule lumen, cytoplasmic vesicle lumen, and vesicle lumen in the CC category (Table 1). Azurophil granules are specialized lysosomes of the neutrophil and contain at least 10 proteins implicated in the killing of microorganisms.^[25,26]^ It may indicate that neutrophils may have higher antimicrobial activity in liver tissues with higher inflammation grades. KEGG pathway analysis revealed that the upregulated DEGs were mainly enriched in Graft-versus-host disease, Rheumatoid arthritis, Leishmaniasis, Influenza A, Type I diabetes mellitus, Hematopoietic cell lineage, Allograft rejection, Natural killer cell-mediated cytotoxicity, Cytosolic DNA-sensing pathway.

MAPK1, ITGA2, CDK2, TGFB1, CDKN2A, MTOR, IL6, PCNA, OAS2, and EP300 were hub genes that had the highest centricity and might play central roles in liver inflammation development. Furthermore, these genes may be potential markers for inflammation development.

This is the first study to use bioinformatics technology to systematically analyze the different expression levels between low grade of liver inflammation and high grade of liver inflammation. Key pathways and genes were identified in the development of hepatic inflammation. The results would bring insight into the mechanism of inflammation development and might provide a theoretical basis and diagnostic markers for HBV-related hepatic inflammation.

There are still limitations in our present study, such as a relatively small sample size and no experimental verification. Therefore, further research investigating the potential mechanisms involved in hepatic inflammation is required.

## 4. Materials and Methods

### 4.1. Data source

Liver tissues were collected from patients with HBV infection, who were hospitalized in the Third Affiliated Hospital of Sun Yat-sen University from 2013 to 2015. Liver tissue samples were divided into 2 groups. The low grade of inflammation group (Group Low) included G0 to G1, 3 samples; the high grade of inflammation group (Group High) included G2 to G4, 9 samples. This grouping method was applied because antiviral treatment was recommended when inflammation grade ≥G2. Arraystar Human LncRNA Microarray V3.0 was designed for the global profiling of human LncRNAs and protein-coding mRNAs, which was updated from the previous Microarray V2.0. About 30,586 LncRNAs and 26,110 coding mRNAs could be detected.

### 4.2. Identification of DEGs

DEGs between Group Low and Group High were identified using R software. Affy, simpleaffy, and plmaffy packages provided in Bioconductor (http://www.bioconductor.org/) were used for quality control, standardization, and log2 conversion for the raw data of microarray.^[27]^ A Limma package was used to screen the DEGs between the 2 groups.^[28]^ The adjusted *P*-value (adj. *P*) was cut by 0.01 and the logFC value was cut by 1.5 with the purpose of providing a balance between the discovery of statistically significant genes and the limitation of false-positive errors. Probe sets without corresponding gene symbols or genes with >1 probe set were removed or averaged, respectively.

### 4.3. KEGG and GO enrichment analyses of DEGs

Clusterprofiler package is a statistical analysis and visualization of functional profiles for genes and gene clusters.^[29]^ The enriched GO categories, including BP, CC, MF, and KEGG pathways were obtained by clusterprofiler package to analyze the differentially expressed genes at the functional level. *P* < .01 was set as the threshold value.

### 4.4. PPI network construction and module analysis

PPI network of DEGs was constructed using the STRINGdb package. Analyzing the functional interactions between proteins might provide insights into the mechanisms of generation and development of the disease. Genes that ranked top 20 in degree, closeness, and betweenness were respectively selected. Genes with degree ≥ 350 were selected as hub genes. PPI network of these hub genes was conducted. Cytoscape (version 3.6.1) is an open source bioinformatics software platform for visualizing molecular interaction networks.^[30]^ The plug-in molecular complex detection (MCODE) of Cytoscape is an APP for clustering a given network based on the topology to find densely connected regions. The most significant modules in the PPI networks were identified using MCODE. The criteria for selection were as follows: MCODE scores > 5, degree cut-off = 2, node score cut-off = 0.2, max depth = 100, and k-core = 4.

## Author contributions

WL conception and design, data analysis and interpretation, and manuscript writing; JYZ collection and/or assembly of data, data analysis and interpretation, and manuscript writing; ZZZ, LYZ collection and/or assembly of data. All authors made substantial revisions to the manuscript draft. All authors have approved the final submitted manuscript.

**Data curation:** Li-Yun Zhao, Zhao-Zhong Zhong.

**Formal analysis:** Li-Yun Zhao.

**Funding acquisition:** Wen Li.

**Methodology:** Wen Li.

**Project administration:** Wen Li.

**Writing – original draft:** Jing-Yuan Zhao, Wen Li.
